# Combining Molecular Subtypes with Multivariable Clinical Models Has the Potential to Improve Prediction of Treatment Outcomes in Prostate Cancer at Diagnosis

**DOI:** 10.3390/curroncol30010013

**Published:** 2022-12-22

**Authors:** Lewis Wardale, Ryan Cardenas, Vincent J. Gnanapragasam, Colin S. Cooper, Jeremy Clark, Daniel S. Brewer

**Affiliations:** 1 Norwich Medical School, University of East Anglia, Norwich Research Park, Norwich NR4 7TJ, UK; 2 Department of Urology, Cambridge University Hospitals NHS Foundation Trust, Cambridge CB2 0QQ, UK; 3 Division of Urology, Department of Surgery, University of Cambridge, Cambridge CB2 0QQ, UK; 4 The Earlham Institute, Norwich Research Park, Norwich NR4 7UZ, UK

**Keywords:** prostate cancer, clinical models, predictive models, molecular subtypes, transcriptome, expression, statistical model

## Abstract

Clinical management of prostate cancer is challenging because of its highly variable natural history and so there is a need for improved predictors of outcome in non-metastatic men at the time of diagnosis. In this study we calculated the model score from the leading clinical multivariable model, PREDICT prostate, and the poor prognosis DESNT molecular subtype, in a combined expression and clinical dataset that were taken from malignant tissue at prostatectomy (*n* = 359). Both PREDICT score (*p* < 0.0001, IQR HR = 1.59) and DESNT score (*p* < 0.0001, IQR HR = 2.08) were significant predictors for time to biochemical recurrence. A joint model combining the continuous PREDICT and DESNT score (*p* < 0.0001, IQR HR = 1.53 and 1.79, respectively) produced a significantly improved predictor than either model alone (*p* < 0.001). An increased probability of mortality after diagnosis, as estimated by PREDICT, was characterised by upregulation of cell-cycle related pathways and the downregulation of metabolism and cholesterol biosynthesis. The DESNT molecular subtype has distinct biological characteristics to those associated with the PREDICT model. We conclude that the inclusion of biological information alongside current clinical prognostic tools has the potential to improve the ability to choose the optimal treatment pathway for a patient.

## 1. Introduction

Prostate cancer is distressingly common (diagnosed in 48,487 of men in UK per year) but not frequently fatal (13% of male cancer deaths) [1]. The progression of prostate cancer is highly heterogeneous [2], and clinical management is challenging [3,4]. It is also estimated, that as many as 50–80% of PSA-detected prostate cancers are clinically irrelevant, that is, even without treatment, they would never have caused any symptoms [5]. This has confounded attempts to develop a consistent and reliable approach to identify aggressive disease. Radical treatment of early prostate cancer, with surgery or radiotherapy, can lead to life changing side-effects of treatment such as impotence or incontinence [6]. There is a need for improved predictors of outcome in non-metastatic men at the time of diagnosis.

One approach is to use the information that is already collected at the point of diagnosis and before treatment, to assess prognosis and the value of treatment. Thurtle et al. (2019) developed an approach, termed ‘PREDICT Prostate’, that modelled, at the time of diagnosis, prostate cancer specific mortality (PCSM) and non-prostate cancer mortality (NPCM) using separate multivariable Cox models within a competing risks framework [7]. The NPCM model utilises the variables age and comorbidity, while the PCSM model combines age, PSA, Gleason grade, clinical T stage, and proportion of positive biopsy cores at the time of diagnosis. The model shows good discrimination in large validation datasets from the UK (*n* = 3000; C-index = 0.84; 95% CI: 0.82–0.86) [7], Singapore (*n* = 2546; C-index = 0.84; 95% CI: 0.80–0.87) [7], Sweden (*n* = 69,206; C-index = 0.85; 95% CI 0.85–0.86) [8], and the United States of America (*n* = 171,942; C-index = 0.82; 95% CI 0.81–0.83) [9]. It has been endorsed by the National Institute for Health and Care Excellence (NICE) [10] and is available in a user-friendly web interface (https://prostate.predict.nhs.uk/ (accessed on 1 May 2022)). Another approach to improve prediction of outcome is to use additional novel biomarkers [11].

Within any single cancer disease type, sub-classification using molecular markers can be an important way to accurately determine prognosis, optimise treatment pathways, and help develop targeted drugs. In previous work, we have successfully identified a novel aggressive molecular subtype of human prostate cancer, called DESNT, that can predict outcome after radical surgery (prostatectomy) and is associated with metastasis. This was discovered by applying the Bayesian clustering method Latent Process Decomposition to transcriptome data [12,13,14]—this takes into account the heterogeneous composition of prostate cancer. Prostatectomy patients with most of their expression assigned to the DESNT type exhibit poor outcomes relative to other patients (*p* < 4.28 × 10^−5^; Log-rank test) and has been validated in eight independent transcriptome datasets. Cancers assigned to the DESNT group have an increased risk of developing metastasis (*X*^2^-test, *p* = 1.86 × 10^−3^) [13]. The amount of the DESNT signature is an independent prognostic predictor of time to biochemical recurrence (HR = 1.52, 95% CI = [1.36, 1.7], *p* = 9.0 × 10^−14^, Cox regression model) [13]. This framework was developed from samples taken at prostatectomy, but we have preliminary data to suggest it’s applicability to biopsies [15]. We are in the process of developing a diagnostic lab to utilise the DESNT framework as an accredited clinical test.

In this work we modelled whether adding the poor prognosis DESNT signature to the PREDICT Prostate algorithm has the potential to improve our ability to predict the overall progress of prostate cancer. Transcriptome data from tumour tissue collected at an initial treatment of proctectomy were used as a proxy for the information that could be gathered from cancerous biopsy tissue at the time of diagnosis. Secondary aims are to determine whether the PREDICT Prostate clinical model can predict disease prognosis after surgical treatment of prostate cancer; and find the similarities and differences in the genes and molecular pathways which drive a higher PREDICT score and characterise the DESNT molecular subtype.

## 2. Materials and Methods

### 2.1. Datasets and Filtering

Microarray datasets from prostate tissue were processed and normalised as described in Luca et al. (2020) (Table 1). In brief, Affymetrix microarray dataset was normalised using the RMA algorithm [16] or previous normalised values were used. Only probes corresponding to genes measured by all platforms were retained. The CamCap and CancerMap datasets have 40 patients in common and thus 20 of the common samples were excluded at random from each dataset. The ComBat algorithm [17] from the sva R package and quantile transformation, was used to mitigate study-specific effects. The ethical approvals obtained for each dataset are listed in the original publications.

The combined dataset was filtered to remove samples that were missing one or more of the clinical variables required for the Prostate PREDICT model (patient’s age, T-stage, PSA and the Gleason histological grade group). Only primary tumour tissue from the prostate were included. Duplicate samples were also removed. For the Stephenson dataset only Gleason sum was available, so 44 samples were removed that had a Gleason sum of seven. The resulting dataset consists of 359 samples.

### 2.2. R Implementation of the Prostate PREDICT Model

The Prostate PREDICT model was originally implemented in the language STRATA [7]. We have translated this to the freely available open-source R statistical programming language [21] and made the code available (https://doi.org/10.5281/zenodo.7248417 accessed on 25 October 2022). Our implementation of the Prostate PREDICT model was extensively verified by comparing the results produced by those of the PREDICT Prostate webpage tool (https://prostate.predict.nhs.uk/tool accessed on 1 May 2022) for a wide variety of inputs. The results were identical, for example, when age = 75, T-stage = 2, PSA = 25 and Gleason score = 4 + 3, the 10-year predicted survival from initial conservative management was 55% via the webpage tool and 0.549 in R. We also examined how the R version PREDICT results vary with clinical variables to ensure that they made logical sense.

As we are interested in reducing radical treatment in prostate cancer the results from the PREDICT model used initial conservative management as the treatment strategy rather than radical treatment. For the datasets used here, comorbidity (the patient had not experienced a hospital admission in the last 2 years for something other than prostate cancer) and detailed biopsy histopathology results were unavailable and so are set to zero, as is done in the online implementation when this information is unavailable. For each sample, the prostate cancer specific mortality probability (PCSM) at ten years after diagnosis (as a percentage) was calculated using as input the associated clinical variables age at diagnosis, PSA at diagnosis, T stage, and prostatectomy Gleason grade group (as a proxy for biopsy Gleason grade group). The non-prostate cancer mortality probability (NPCM, as a percentage) was calculated using age at diagnosis. The PREDICT score, the increase in probability of mortality at 10 years from having prostate cancer, was defined as NPCM-PCSM.

### 2.3. DESNT Score and Assignment

Latent Process Decomposition (LPD) was applied to the MSKCC dataset [18] to produce the DESNT framework model, exactly as described in Luca et al. (2020) [13]. This model was then applied to the other datasets using the OAS-LPD algorithm, a modified version of the LPD algorithm in which new sample(s) are decomposed into LPD signatures, without retraining the model. Again, as described in Luca et al. (2020). LPD is an unsupervised Bayesian approach which decomposes each sample’s expression into signature expression profiles of each molecular subtype. For each sample a score between 0 and 1 is given for each subtype which represents the proportion of a sample’s expression that is explained by the signature expression profile for that subtype. Here, the proportion of expression assigned to the DESNT subtype is termed the DESNT score and are the exact scores produced in previous work [13]. If the DESNT score is the largest score across the subtypes, the sample is considered a member of the DESNT subtype.

### 2.4. Differential Gene Expression Analysis

Differentially expressed genes were identified for each comparison using a moderated *t*-test implemented in the limma (v 3.52.0) R package [22] with a threshold of Benjamin-Hochberg false discovery rate < 0.05.

### 2.5. Functional Enrichment Analysis

Functional enrichment analysis was performed using the gProfiler2 (v0.2.1) [23] R package utilising the KEGG, RECTOME, and Gene Ontology database for biological process terms. The gSCS (Set Counts and Sizes) correction method was used to determine significantly enriched pathways and ontology terms with significance *p* < 0.05.

### 2.6. Statistical Tests

All analyses were performed in R (version 4.1.2) using default parameters unless otherwise stated. Survival analyses were performed using Cox proportional hazards regression models, the log-rank test, and Kaplan–Meier estimator, as implemented in the survival R package with biochemical recurrence after prostatectomy as the end point. Pairwise comparisons of Kaplan–Meier curves using Log-Rank test were performed using the SurvMiner (v 0.4.9), with *p*-values adjusted using the Benjamini-Hochberg multiple testing correction. All plots were created using ggplot2 (v 3.3.6). All statistical tests performed were two-sided non-parametric tests unless otherwise stated.

## 3. Results

### 3.1. Data Overview

We combined transcriptome data from malignant samples taken at an initial treatment of prostatectomy from four studies: the MSKCC [18], CancerMap [12], Stephenson [19] and CamCap [20] studies (Table 1). These were filtered to have results from one primary sample per patient with the required clinical information required for the Prostate PREDICT model (*n* = 359; Table 2). The proportion of expression assigned to the DESNT poor prognosis molecular subtype (DESNT score) were gathered from previous results [13]. For each sample, the prostate cancer specific mortality probability (PCSM) at ten years after diagnosis (as a percentage) was calculated using an implementation of the Prostate PREDICT model in R (see methods), under the assumption of initial conservative management, using as input the associated clinical variables: age at diagnosis, PSA at diagnosis, clinical T stage, and prostatectomy Gleason grade group (as a proxy for biopsy Gleason grade group). The equivalent expected non-prostate cancer mortality (NPCM) was calculated using age at diagnosis. The PREDICT score, the increase in probability of mortality at 10 years caused from having prostate cancer, was defined as NPCM-PCSM.

**Figure 1:**
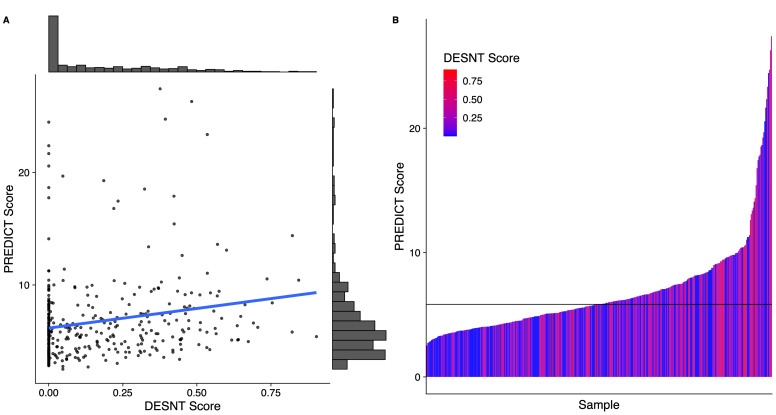
The relationship between Prostate PREDICT score and DESNT score. (**A**) Scatter plot and distribution. (**B**) A waterfall plot showing how the DESNT score varies with PREDICT score. The horizontal line at 5.8 represents the median PREDICT score. The PREDICT score is the percentage increase in probability of mortality at 10 years from having prostate cancer defined as the non-prostate cancer mortality minus prostate cancer specific mortality at 10 years. DESNT score is the proportion of expression that is explained by the signature expression profile of the DESNT molecular subtype.

**Figure 2:**
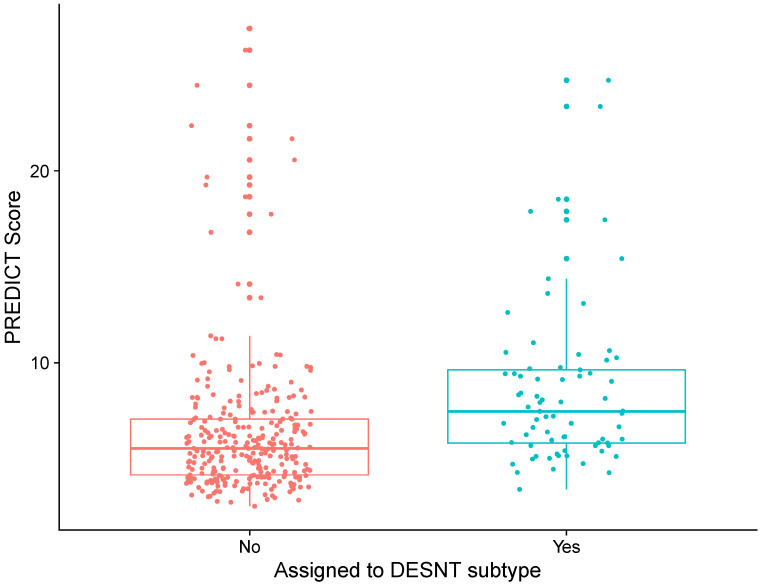
Differences in PREDICT score between prostate cancer samples assigned to DESNT and those not. Samples assigned to DESNT are those where the largest proportion of expression is explained by the expression signature of the DESNT subtype. The PREDICT score is the increase in probability of mortality at 10 years caused by having prostate cancer defined as the non-prostate cancer mortality minus prostate cancer specific mortality at 10 years.

DESNT scores from our combined dataset had a median value of 0.09 and an interquartile range of 0.32. PREDICT scores had a median value of 5.84 and an interquartile range of 3.24. There was a weak correlation between DESNT score and PREDICT score (Figure 1A; rho = 0.21; *p* < 0.05; Spearmen’s correlation). The DESNT score is very variable with respect to the PREDICT score (Figure 1B). The PREDICT score showed a statistically significant increase in samples that were DESNT cancers, i.e., where the proportion assigned to the DESNT subtype was higher than all other subtypes in the framework (Figure 2; *p* < 0.001; Mann–Whitney U test; difference in medians = 1.93).

### 3.2. Predictive Ability of PREDICT and DESNT Score to Predict Time to Biochemical Recurrence

**Figure 3:**
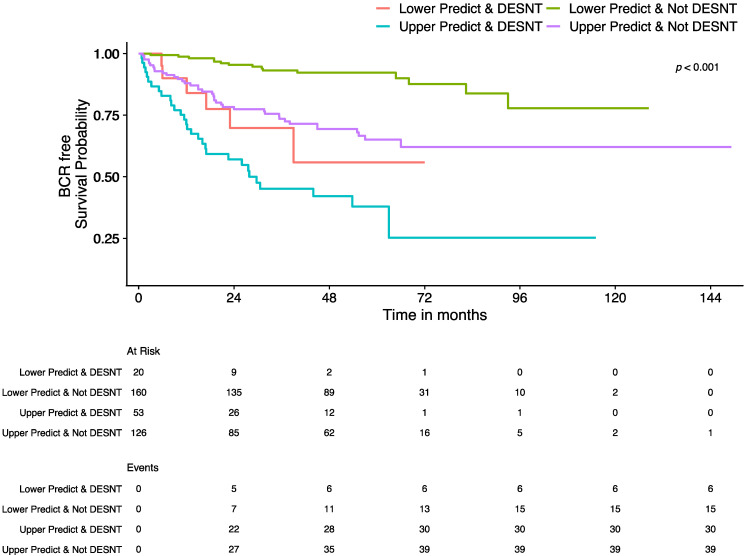
Kaplan–Meier plot showing the survival curves for samples grouped by DESNT and Prostate PREDICT model status. Endpoint is time to biochemical recurrence (BCR). Samples are divided into DESNT vs. non-DESNT and upper PREDICT vs. lower PREDICT (split around the median PREDICT score). The “At Risk” table below the plot shows the number of patients in each group, at the corresponding time point, that have not had a biochemical recurrence event and have longer follow up than that time. The “Events” table shows the cumulative number of biochemical recurrence events observed in a group at that time point.

Both PREDICT score and DESNT score, when applied in separate models, have a significant association with time to biochemical recurrence (PREDICT: *p* < 0.0001, IQR HR = 1.59 [95% CI 1.43–1.76]; DESNT: *p* < 0.0001, IQR HR = 2.08 [95% CI 1.58–2.76]; Cox proportional hazards regression). A joint Cox proportional hazards model built with the two continuous independent variables PREDICT and DESNT score (*p* < 0.0001, IQR HR = 1.53 and 1.79, respectively; Table 3) was significantly better at predicting biochemical recurrence outcome than PREDICT score (*p* < 0.001; likelihood ratio test) or DESNT score (*p* < 0.001) alone. To illustrate this, samples were categorised into DESNT cancers or non-DESNT cancers, and upper PREDICT score or lower PREDICT score (split around the median; Table 4). A Kaplan-Meir plot shows clear delineation between each combination of groups (Figure 3; Log-rank *p*-value < 0.001). At five years, the estimated proportion that are biochemical recurrence free are 92% (Lower PREDICT score & Not DESNT), 65% (Upper PREDICT score & Not DESNT), 56% (Lower PREDICT score & DESNT), and 38% (Upper PREDICT score & DESNT). Pair-wise, all Kaplan–Meier curves are significantly different (*p* < 0.001; log-rank test; Benjamini-Hochberg adjusted *p*-values) apart from “Lower PREDICT score & DESNT” vs. “Upper PREDICT score & DESNT” (*p* = 0.18) and “Lower PREDICT score & DESNT” vs. “Upper PREDICT score & Not DESNT” (*p* = 0.53).

### 3.3. Characterisation of the Genes and Biological Processes behind PREDICT and DESNT Scores

To biologically characterise the PREDICT score we compared the expression profiles from samples with the top 25% PREDICT score versus the bottom 25%. We found 451 genes to be significantly differentially expressed (287 downregulated; 164 upregulated; adjusted *p*-values < 0.05; adapted *t*-test; Table 5; Table S1). 162 pathways or ontological terms were found to be significantly enriched in upregulated genes and 74 with downregulated genes (*p* < 0.05; Table S2). This corresponded to 63 GO biological process terms, six KEGG pathways, and five Reactome pathways for downregulated genes, and 143 GO biological process terms, four KEGG pathways, and 15 Reactome pathways for downregulated genes (see Table 6 for enriched Reactome pathways).

4507 genes were found to be differentially expressed between DESNT vs. non-DESNT samples (1973 downregulated; 2534 upregulated; adjusted *p*-values < 0.05; adapted *t*-test; Table 7; Table S3). Of the 51 differential expressed genes Luca et al. (2020) observed as characteristic of the DESNT subtype across multiple datasets (49 down-regulated, two up-regulated), all of them were differentially expressed in this analysis and were altered in the same direction (Table S3). A much larger number of genes were identified here as the DESNT characteristic genes reported in Luca et al. are the overlap of differentially expressed genes from multiple different comparisons in independent cohorts. 449 pathways or ontological terms were found to be significantly enriched in upregulated genes and 1391 with downregulated genes (*p* < 0.05; Table S4). This corresponded to 1288 GO biological process terms, 58 KEGG pathways, and 45 Reactome pathways for downregulated genes, and 373 GO biological process terms, nine KEGG pathways, and 67 Reactome pathways for upregulated genes (see Table 8 for the top 10 enriched Reactome pathways).

Of the 451 genes found to be characteristic of PREDICT, the majority (78%) were also found to be differentially expressed in the same direction between DESNT vs. non-DESNT samples (Table S1), but only 8% of DESNT differentially expressed genes were found to be differentially expressed in the same direction between PREDICT score high vs. PREDICT score low samples. Only 24 out of 51 characteristic DESNT genes from Luca et al. (2020) were found to characterise PREDICT. 93 out of 236 (40%) enriched pathways/ontology terms were unique to the PREDICT Score (Table S2; 37% GO biological process terms, 80% KEGG pathways and 40% Reactome pathways). Similarly, 1697 out of 1840 (92%) enriched pathways/ontology terms were unique to DESNT (Table S4; 92% GO biological process terms, 97% KEGG pathways and 89% Reactome pathways). Taken together these results are suggestive that DESNT provides additional information to PREDICT based on the underlying biological processes.

## 4. Discussion

In this study we examined four large expression data sets that were taken from primary prostate cancer samples at prostatectomy for men with prostate cancer that had not received any other treatment. This data, along with relevant clinical data, was used as a proxy of the biological information that could be gathered from biopsies at the time of diagnosis. We calculated the model score from the leading clinical multivariable model, PREDICT prostate, and the poor prognosis DESNT molecular subtype. We showed the potential for the PREDICT Prostate clinical model to predict disease prognosis after surgical treatment of prostate cancer. We also found that by combining the DESNT score with the PREDICT score produced a significantly better predictor of outcome following prostatectomy. The return of prostate cancer after prostatectomy is an indication that micrometastases were present at the time of surgery [24]—it is estimated that up to 70% of patients have disseminated tumour cells after prostatectomy [25]. Therefore, poor treatment response at prostatectomy may give an indication of overall disease state. Our findings are important because it suggests that we can make a better-informed decision at the time of diagnosis of whether to perform radical treatment or not if molecular information is included.

For the first time the biological mechanisms behind an increased probability of mortality at ten years after diagnosis caused by prostate cancer (i.e., a higher PREDICT score) has been examined. The top 10 differentially expressed genes are the downregulation of *ANPEP*, *CD38*, *SLC22A3*, *NPY*, *MSMB*, *MT1G*, *MT1M*, *KRT15*, & *SFRP4* and upregulation of *COMP*.

*ANPEP* was the top-ranked downregulated gene in both PREDICT and DESNT analyses. Aminopeptidase N (APN) is the enzyme encoded by *ANPEP* that belongs to a group of widely expressed ectopeptidases [26]. *APN* is multifunctional for the post-secretory processing of neuropeptides and regulating the access of these molecules to cellular receptors. The role of *APN* positively associated with intracellular signalling and has been shown to play an important role in metastasis of several malignancies, including prostate cancer through neoangiogenesis [27,28,29]. Sorenson et al. (2013) observed a significant (*p* < 0.001) downregulation of *ANPEP* expression in prostate cancer in comparison with non- malignant prostate tissue samples [30]. The authors concluded that negative *APN* immunoreactivity is a prognostic factor for patients harbouring clinically localised prostate cancer for both recurrence-free and cancer-specific survival endpoints.

CD38 has previously been reported as a marker of the luminal cells in human prostate [31]. Using CD38 as a marker, Liu et al. (2016) identified low expression of the gene in a progenitor-like subset of luminal cells within the human prostate that are capable of initiation of human prostate cancer in an in vivo tissue-regeneration assay [32]. They also demonstrated that luminal cells with low CD38 expression are associated with disease progression and poor survival outcome in prostate cancers.

Neuropeptide Y (*NPY*) is a gene involved in various physiological and homeostatic processes such as stress response. Liu et al. (2007) observed lower expression levels of *NPY* that were associated with more aggressive clinical behaviour in prostate cancer [33]. *MT1G* [34], *MSMB* [35], *SLC22A3* [36], *COMP* [37] and *KRT15* [38] have also been associated with aggressive and/or poor clinical outcome in prostate cancer.

Functional enrichment analysis identified many molecular pathways that were upregulated or downregulated in high PREDICT score samples. This included the upregulation of many cell cycle related pathways, a well-known hallmark of cancer [39] and the downregulation of metabolism and cholesterol biosynthesis. Consistent with this result, Rye et al. found robust and consistent downregulation of nearly all genes in the cholesterol synthesis pathway in prostate cancer [40].

In this study we have also shown that the DESNT molecular subtype has shared and distinct biological characteristics to the general aggressive phenotype picked up by the PREDICT prognosis model. A much larger number of differentially expressed genes and enriched pathways were detected. This suggests that samples assigned as DESNT have expression profiles that are more like each other than samples with similar PREDICT scores, and so there is greater statistically power to detect differences. Only 8% of DESNT differentially expressed genes were found to be differentially expressed in the same direction in PREDICT score high samples. There were also many distinct enriched pathways including the downregulation of signalling pathways and extracellular matrix organisation and upregulation of DNA replication and translation. The DESNT signature has a distinctive biological profile, which is further evidence that it is a valid molecular subtype.

This study has several limitations. Firstly, data comes from prostatectomy samples rather than biopsy samples at diagnosis. This confines the characteristics of the cohort and often Gleason score is upgraded at prostatectomy [41], although prostatectomy was the primary treatment closest to diagnosis for these patients and so is a reasonable proxy to use. Secondly, the full power of the PREDICT model could not be utilised as full diagnostic biopsy information was unavailable for these datasets. Thirdly, biochemical recurrence was used as the clinical endpoint whereas metastatic disease or cancer-specific death would be more informative—PREDICT was not developed or calibrated for biochemical relapse as an outcome hence its performance in this setting has not previously been assessed. Finally, compared to the tens of thousands that the PREDICT model has been validated in, the numbers are relatively low, however we have used robust methods to compensate for this and reported confidence intervals throughout. Despite these limitations, the results support the notion of the potential value of including biological measurements along with the clinical variables collected as part of the standard clinical pathway. Future studies where transcriptome data is generated from a large series of biopsies with good quality clinical data with long follow up would be welcomed.

There is a need for improved predictors of outcome in non-metastatic men at the time of diagnosis to allow the optimal treatment pathway to be chosen. The inclusion of biological information, in particular the DESNT poor prognosis molecular subtype, alongside the best-of-breed clinical prognostic tool, PREDICT prostate, has the potential to make this improvement. This combination has the potential to help avoid unnecessary treatments and life-altering side-effects and improve survival in prostate cancer patients.

## Figures and Tables

**Table 1:** Transcriptome datasets used. FF = Fresh Frozen.

Dataset	Primary	Normal	Type	Platform	Citation
MSKCC [18]	131	29	FF	Affymetrix Exon 1.0 ST v2	Taylor et al., 2010
CancerMap [12]	137	17	FF	Affymetrix Exon 1.0 ST v2	Luca et al., 2018
Stephenson [19]	78	11	FF	Affymetrix U133A	Stephenson et al., 2005
CamCap [20]	147	73	FF	Illumina HT12 v4.0 BeadChip	Ross-Adams et al., 2015

**Table 2:** Summary of clinical variables of cohorts. BCR = Biochemical recurrence after prostatectomy as defined by two PSA measurements at values greater than or equal to 0.2 ng/mL. BCR/Follow up is time to biochemical recurrence or last clinical update. T-stage = clinical tumour stage. The PREDICT score, the percentage increase in probability of mortality at 10 years from having prostate cancer, is defined as the non-prostate cancer mortality minus prostate cancer specific mortality at 10 years. DESNT score is the proportion of expression assigned to the DESNT poor prognosis molecular subtype.

Characteristic	CamCap, *n* = 89 ^1^	CancerMap, *n* = 108 ^1^	Stephenson, *n* = 33 ^1^	MSKCC, *n* = 129 ^1^
Age at diagnosis	61 (56, 65)	62 (56, 65)	61 (55, 65)	58 (54, 62)
PSA at diagnosis (ng/mL)	7.9 (6.1, 9.8)	7.9 (5.8, 11.4)	9.8 (6.0, 18.4)	5.9 (4.5, 9.3)
Gleason grade group				
1	12 (13%)	29 (27%)	15 (45%)	40 (31%)
2	52 (58%)	59 (55%)	0 (0%)	53 (41%)
3	16 (18%)	16 (15%)	0 (0%)	21 (16%)
4	8 (9.0%)	1 (0.9%)	10 (30%)	8 (6.2%)
5	1 (1.1%)	3 (2.8%)	8 (24%)	7 (5.4%)
T Stage				
1	48 (54%)	1 (0.9%)	19 (58%)	0 (0%)
2	28 (31%)	58 (54%)	13 (39%)	84 (65%)
3	13 (15%)	49 (45%)	1 (3.0%)	39 (30%)
4	0 (0%)	0 (0%)	0 (0%)	6 (4.7%)
BCR/Follow up (in months)	23 (15, 41)	55 (32, 64)	56 (12, 70)	47 (28, 62)
BCR event				
FALSE	74 (83%)	77 (71%)	16 (48%)	102 (79%)
TRUE	15 (17%)	31 (29%)	17 (52%)	27 (21%)
DESNT Score	0.22 (0.10, 0.37)	0.09 (0.00, 0.31)	0.10 (0.02, 0.34)	0.00 (0.00, 0.18)
PREDICT Score	5.5 (4.7, 7.0)	6.4 (5.0, 8.1)	7.7 (3.6, 10.4)	5.6 (4.2, 7.2)

^1^ Median (IQR); *n* (%)

**Table 3:** Summary of Cox proportional hazard model combining PREDICT score and DESNT score. Endpoint is time to biochemical recurrence.

Variable	IQR Hazard Ratio (HR)	HR Lower 95% CI	HR Upper 95% CI	*p*-Value
PREDICT Score	1.53	1.37	1.70	<0.0001
DESNT score	1.79	1.34	2.40	<0.0001

**Table 4:** Discretisation of samples into DESNT vs. non-DESNT (based on the dominant subtype expression signature) and upper and lower PREDICT score (split around the median PREDICT score).

	DESNT	Non-DESNT
Upper PREDICT	53	126
Lower PREDICT	20	160

**Table 5:** The top ten differentially expressed genes between the samples with the 25% highest PREDICT scores versus the lowest 25% PREDICT scores, ranked by log_2_ fold change. *p* values adjusted for multiple testing correction using the Benjamini-Hochberg algorithm. Whether these genes overlap with the 51 significant differential expressed genes Luca et al. (2020) observed as characteristic of the DESNT subtype and the differentially expressed genes for DESNT found in this study are also shown. Full results in Table S1.

Gene Symbol	log_2_ Fold Change	*p* Value	Adjusted *p* Value	Overlap with Luca et al. DESNT Genes	Overlap with DESNT DEGs
*ANPEP*	−1.31	1.54 × 10^−8^	1.33 × 10^−5^	TRUE	TRUE
*CD38*	−1.02	1.25 × 10^−9^	2.17 × 10^−6^	FALSE	TRUE
*SLC22A3*	−0.95	7.94 × 10^−12^	8.28 × 10^−8^	FALSE	TRUE
*NPY*	−0.91	2.84 × 10^−4^	1.32 × 10^−2^	FALSE	FALSE
*MSMB*	−0.89	1.22 × 10^−5^	1.84 × 10^−3^	FALSE	TRUE
*MT1G*	−0.86	4.51 × 10^−7^	1.54 × 10^−4^	FALSE	TRUE
*MT1M*	−0.85	8.34 × 10^−6^	1.32 × 10^−3^	TRUE	TRUE
*COMP*	0.84	8.81 × 10^−9^	1.02 × 10^−5^	FALSE	TRUE
*KRT15*	−0.77	1.66 × 10^−5^	2.25 × 10^−3^	FALSE	TRUE
*SFRP4*	0.76	2.39 × 10^−8^	1.66 × 10^−5^	FALSE	TRUE

**Table 6:** Reactome pathways found to be significantly enriched for significantly upregulated or downregulated genes for PREDICT high score samples versus low score samples. Direction = whether pathway is enriched for downregulated or upregulated genes. Full results including KEGG pathway and GO BP terms in Table S2.

Term Name	Direction	*p* Value	Term Size	Intersection Size	Overlap with DESNT Enriched Terms
Cell Cycle, Mitotic	Up	4.08 × 10^−9^	548	28	TRUE
Cell Cycle	Up	2.37 × 10^−8^	678	30	TRUE
Mitotic G1 phase and G1/S transition	Up	3.79 × 10^−5^	147	12	TRUE
Integrin cell surface interactions	Up	0.002	84	8	FALSE
G1/S-Specific Transcription	Up	0.005	27	5	TRUE
Mitotic Prometaphase	Up	0.006	199	11	TRUE
G1/S Transition	Up	0.006	130	9	TRUE
ECM proteoglycans	Up	0.008	75	7	FALSE
Cell Cycle Checkpoints	Up	0.009	290	13	TRUE
Mitotic Spindle Checkpoint	Up	0.012	109	8	TRUE
M Phase	Up	0.021	407	15	TRUE
Kinesins	Up	0.023	60	6	FALSE
Resolution of Sister Chromatid Cohesion	Up	0.030	123	8	TRUE
Amplification of signal from unattached kinetochores via a MAD2 inhibitory signal	Up	0.032	92	7	TRUE
Amplification of signal from the kinetochores	Up	0.032	92	7	TRUE
Metabolism	Down	0.000	2075	63	FALSE
Cholesterol biosynthesis	Down	0.001	24	6	FALSE
Glutathione conjugation	Down	0.009	34	6	FALSE
Response to metal ions	Down	0.031	14	4	FALSE
Metabolism of lipids	Down	0.046	728	27	FALSE

**Table 7:** The top ten differentially expressed genes between the samples classified as DESNT samples versus non-DESNT samples, ranked by log_2_ fold change. *p* values adjusted for multiple testing correction using the Benjamini-Hochberg algorithm. Whether these genes overlap with the 51 significant differential expressed genes Luca et al. (2020) observed as characteristic of the DESNT subtype and the differentially expressed genes for PREDICT score found in this study are also shown. Full results in Table S3.

Gene Symbol	log_2_ Fold Change	*p* Value	Adjusted *p* Value	Overlap with Luca et al. DESNT Genes	Overlap with PREDICT DEGs
*ANPEP*	−2.27	4.18 × 10^−31^	2.18 × 10^−27^	TRUE	TRUE
*RLN1*	−1.80	4.50 × 10^−14^	3.19 × 10^−12^	FALSE	FALSE
*MT1M*	−1.62	1.45 × 10^−25^	3.79 × 10^−22^	TRUE	TRUE
*ALOX15B*	−1.44	2.85 × 10^−17^	4.95 × 10^−15^	FALSE	TRUE
*CD38*	−1.43	1.99 × 10^−23^	1.59 × 10^−20^	FALSE	TRUE
*MSMB*	−1.42	5.48 × 10^−14^	3.69 × 10^−12^	FALSE	TRUE
*MT1G*	−1.41	3.30 × 10^−19^	1.04 × 10^−16^	FALSE	TRUE
*F5*	1.35	1.06 × 10^−19^	3.56 × 10^−17^	TRUE	TRUE
*LEPREL1*	−1.33	1.85 × 10^−23^	1.59 × 10^−20^	FALSE	TRUE
*ACTG2*	−1.33	9.88 × 10^−23^	6.87 × 10^−20^	TRUE	TRUE
*ERG*	1.31	2.26 × 10^−12^	9.90 × 10^−11^	FALSE	FALSE

**Table 8:** The top 10 Reactome pathways found to be uniquely significantly enriched for significantly upregulated or downregulated genes for DESNT sample status, ranked by significance. Direction = whether pathway is enriched for downregulated or upregulated genes. Full results in Table S4.

Term Name	Direction	*p* Value	Term Size	Intersection Size
Signal Transduction	Down	3.94 × 10^−15^	2523	437
Platelet activation, signaling and aggregation	Down	3.91 × 10^−11^	258	76
Metabolism of RNA	Up	6.61 × 10^−11^	661	173
Translation	Up	1.68 × 10^−10^	292	94
Response to elevated platelet cytosolic Ca2+	Down	3.61 × 10^−9^	130	46
Signaling by Receptor Tyrosine Kinases	Down	3.99 × 10^−9^	502	115
Platelet degranulation	Down	1.27 × 10^−8^	125	44
Hemostasis	Down	3.12 × 10^−8^	672	140
DNA Replication	Up	3.40 × 10^−8^	128	50
Extracellular matrix organization	Down	3.81 × 10^−8^	298	77

## Data Availability

The datasets analysed during the current study are publicly available: MSKCC [18]: https://www.ncbi.nlm.nih.gov/geo/query/acc.cgi?acc=GSE21034 (accessed on 1 May 2016); CancerMap [12]: https://www.ncbi.nlm.nih.gov/geo/query/acc.cgi?acc=GSE94767 (accessed on 1 May 2016); Stephenson [19]: Data available from the corresponding author of this paper. CamCap [20]: https://www.ncbi.nlm.nih.gov/geo/query/acc.cgi?acc=GSE70768 (accessed on 1 May 2016) and https://www.ncbi.nlm.nih.gov/geo/query/acc.cgi?acc=GSE70769 (accessed on 1 May 2016).

## Supplementary Materials

The following supporting information can be downloaded at: https://www.mdpi.com/article/10.3390/curroncol30010013/s1, Table S1: Significantly differentially expressed genes between the samples with the 25% highest PREDICT scores versus the lowest 25% PREDICT scores; Table S2: KEGG pathways, Reactome pathways, and Gene Ontology biological processes terms found to be significantly enriched for significantly upregulated or downregulated genes for PREDICT high score samples versus low score samples; Table S3: Significantly differentially expressed genes between the samples classified as DESNT samples versus non-DESNT samples; Table S4: KEGG pathways, Reactome pathways, and Gene Ontology biological processes terms found to be significantly enriched for significantly upregulated or downregulated genes for DESNT sample status.
